# Comparison of the effect of gas and silicone oil tamponades on visual outcomes in the surgical treatment of rhegmatogenous retinal detachment

**DOI:** 10.1186/s40942-026-00851-0

**Published:** 2026-04-07

**Authors:** Tural Galbinur, Ayan Mammadkhanova, Hafiz Gahramanov, Ulkar Galbinur, Ayan Galbinur, Gunay Aslanova, Pasha Musayev

**Affiliations:** 1 Department of Ophthalmology, Azerbaijan Medical University, Baku, Azerbaijan; 2 Ophthalmo Retina Center, National Prime Hospital, Baku, Azerbaijan

**Keywords:** Retinal detachment, Silicone oils, Gas tamponade, Vitrectomy

## Abstract

**Purpose:**

The choice of tamponade material for pars plana vitrectomy during rhegmatogenous retinal detachment surgery determines the overall success of the procedure. This study examined how gas and silicone oil tamponades affect visual results, retinal thickness measurements, and treatment-related complications.

**Methods:**

Records of patients who underwent vitrectomy for primary rhegmatogenous retinal detachment in our clinic between January 2020 and December 2025 were retrospectively reviewed. A total of 113 eyes were included in the study, of which 68 were treated with gas and 45 with silicone oil tamponade. Propensity score matching was performed to eliminate baseline differences between groups, with a balance target of standardized mean difference < 0.20 for all covariates, and 40 patients in each group were analyzed. Visual acuity, intraocular pressure, retinal thickness parameters on optical coherence tomography, and complications were evaluated.

**Results:**

At six months, the median BCVA was 0.18 (IQR 0.10–0.40) logMAR in the gas group and 0.48 (IQR 0.22–0.90) logMAR in the silicone oil group (*p* = 0.003; mean: 0.32 ± 0.49 vs. 0.64 ± 0.59 logMAR), with 70% of silicone oil eyes still having oil in situ at this time point. Post-tamponade clearance BCVA remained superior in the gas group (median 0.18 vs. 0.42 logMAR, *p* = 0.038). Clinically significant visual improvement was achieved in the gas group 70% and silicone oil group 47.5%. A significant decrease in parafoveal retinal thickness, including RNFL, GCL, and GCL + IPL layers, and increase in intraocular pressure were observed in the silicone oil group. Post-tamponade clearance anatomical success rates were similar in both groups (95.0% vs. 87.5%, *p* = 0.432). In the multivariate analysis, tamponade type was found to be an independent factor affecting visual outcomes.

**Conclusion:**

Gas tamponade provides better functional outcomes than silicone oil in the treatment of primary rhegmatogenous retinal detachment. In addition to anatomical success, long-term visual prognosis and the potential for inner retinal layer toxicity should also be considered when selecting tamponade.

## Introduction

Rhegmatogenous retinal detachment (RRD) is a medical condition that leads to permanent deterioration of vision when left untreated and requires immediate surgical treatment. Surgical treatment for primary RRD involves pars plana vitrectomy (PPV) as the main procedure, while gas and silicone oil tamponades function as conventional treatment methods [1, 2]. The natural absorption of gas tamponade occurs, but silicone oil tamponades provide prolonged retinal stability during complex treatment situations [3]. The anatomical success rates of gas and silicone oil tamponades for retinal recovery show comparable results, although they produce different effects on visual performance [1, 4]. Studies using optical coherence tomography (OCT) have demonstrated that silicone oil treatment results in retinal thinning and reduced choroidal thickness and microvascular injury. The application of silicone oil tamponade results in three major complications: unexplained vision decline, cystic macular edema, and permanent damage to the retinal tissue [5, 6].

Research findings on this topic show conflicting results because the studies examined different groups of patients over short follow-up periods. However, the current understanding of how silicone oil affects retinal structure and visual function remains unclear [7, 8]. The research findings about retinal thinning and cystic edema reversal show inconsistent results. We hypothesized that gas tamponade would yield superior visual outcomes compared to silicone oil tamponade in the surgical management of primary RRD.

## Material and methods

### Patients and clinical evaluation

**Fig. 1:**
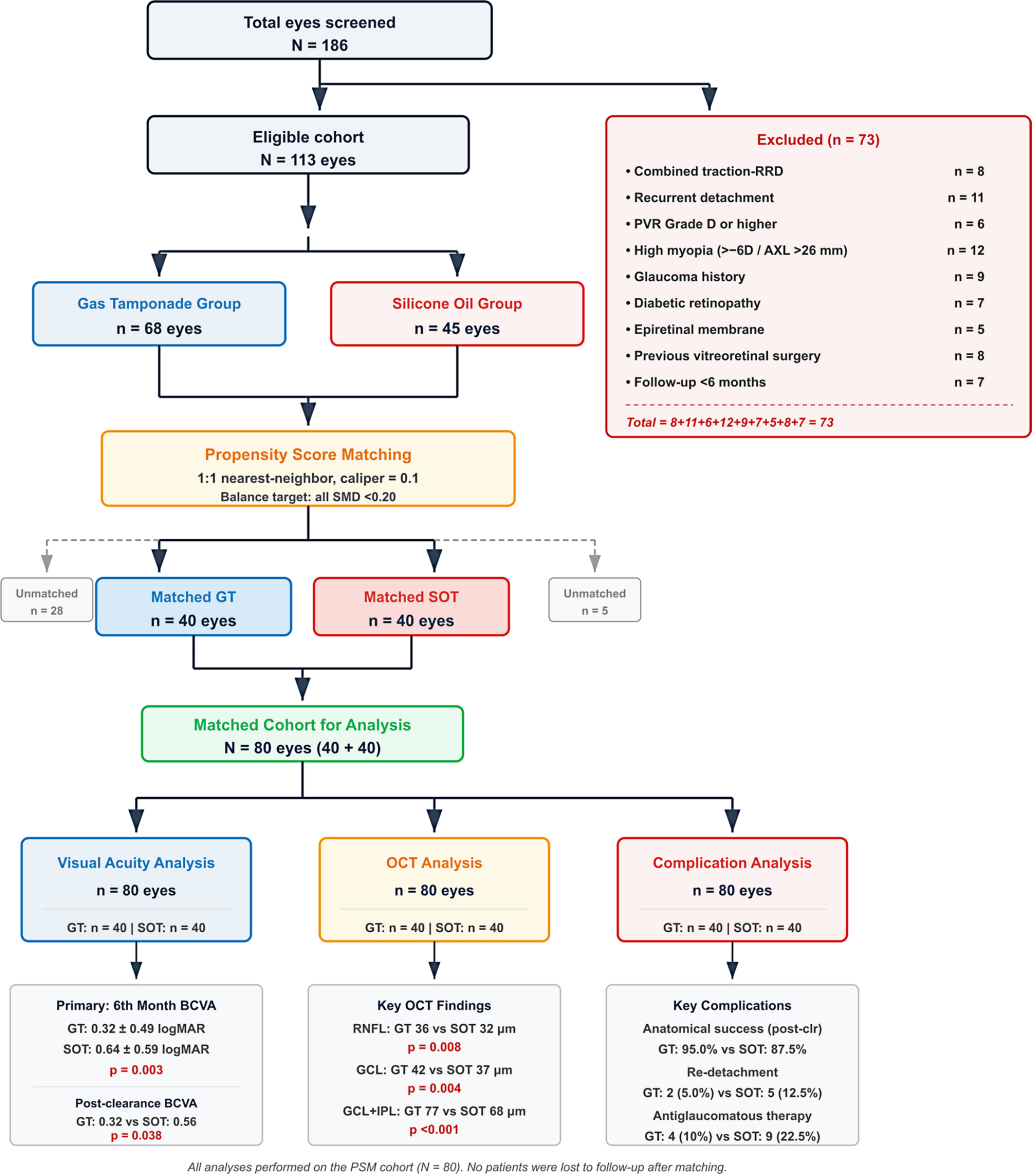
Patient flow diagram. RRD: rhegmatogenous retinal detachment; PVR: proliferative vitreoretinopathy; AXL: axial length; PSM: propensity score matching; SMD: standardized mean difference; GT: gas tamponade; SOT: silicone oil tamponade; BCVA: best-corrected visual acuity; RNFL: retinal nerve fiber layer; GCL: ganglion cell layer; IPL: inner plexiform layer

This retrospective cohort study was conducted between January 2020 and December 2025 by reviewing the records of patients who underwent pars plana vitrectomy (PPV) for primary rhegmatogenous retinal detachment (RRD). The sample size was calculated with reference to the effect size reported in similar studies (Cohen’s d: 0.65). As a result of the power analysis, it was determined that there should be at least 40 patients in each group, with 80% statistical power and 0.05 alpha error. Patients aged > 18 years who underwent retinal detachment surgery for the first time and completed at least six months of postoperative follow-up were included in the study [9]. The minimum follow-up criterion of 6 months applied to the entire postoperative observation period. In the silicone oil group, the follow-up period commenced at surgery and extended through and beyond silicone oil removal; all patients in the silicone oil tamponade group were followed for a minimum of 3 months after silicone oil removal, which itself occurred at a mean of 7.8 ± 3.2 months postoperatively. Therefore, the minimum total follow-up for silicone oil-treated patients exceeded 6 months in all cases. The overall follow-up period ranged from 6 to 14 months (median 7 months) in the gas tamponade group and from 7 to 18 months (median 11 months) in the silicone oil group. Patients with combined tractional–rhegmatogenous detachment, recurrent detachment, proliferative vitreoretinopathy (PVR) of stage D and above, high myopia (greater than minus 6 diopters or axial length greater than 26 millimeters), history of glaucoma, diabetic retinopathy, and epiretinal membrane, and those with a history of previous vitreoretinal surgery were excluded from the study [5]. Complex RD was defined as three or more quadrant involvement, PVR Grade B and above, giant tear, or choroidal detachment. Visual acuity measured using the Snellen chart in clinical evaluations was converted to logMAR units for statistical analysis [5]. Intraocular pressure, axial length, retinal thickness, and retinal volume were recorded in standard units. Patient selection and the flow of participants through the study are summarized in (Fig. 1).

### Clinical procedures and imaging

In the preoperative evaluation, prognostic parameters such as symptom duration, lens status, macular involvement, extent of detachment, degree of PVR, presence of choroidal detachment, and presence of giant tears were recorded, in addition to the demographic data of the patients [9]. Visual acuity and intraocular pressure measurements were repeated preoperatively and at postoperative 1st, 3rd, and 6th months [9]. Retinal imaging was performed with an optical coherence tomography (OCT) device (Zeiss Cirrus 5000 HD-OCT, Germany) [5]. OCT analyses evaluated central macular thickness (CMT), parafoveal quadrant thickness (nasal, temporal, superior, and inferior), retinal nerve fiber layer (RNFL) thickness, ganglion cell layer (GCL) thickness, ganglion cell–inner plexiform layer (GCL + IPL) complex thickness, inner retinal thickness, total retinal thickness, retinal volume, and subfoveal choroidal thickness parameters [5]. Qualitative OCT assessment included foveal contour flattening, ellipsoid zone integrity, inner segment/outer segment (IS/OS) band continuity, RNFL wedge-shaped defects, and the presence of subfoveal fluid or cystoid changes. In macula-off cases, preoperative OCT acquisition was performed in all patients despite the presence of subretinal fluid. Scans with poor signal quality (signal strength index < 6) were excluded from analysis. In macula-off eyes, central foveal parameters such as CMT should be interpreted with caution due to subretinal fluid-related optical distortion; however, parafoveal quadrant thicknesses and inner retinal layer measurements (RNFL, GCL, GCL + IPL) obtained outside the area of maximal detachment remained technically feasible and interpretable. The presence of subretinal fluid as a confounding factor for preoperative foveal OCT measurements is acknowledged as a study limitation (see also Limitations). Measurements in the silicone oil applied group were performed in three stages: preoperatively, with silicone oil in place, and at least three months after removal. In the gas group, OCT measurements were obtained preoperatively and at 6 months postoperatively (gas fully absorbed by 6–8 weeks). Anatomical success was defined as attachment of the retina without any tamponade remaining in the eye, assessed at 6 months postoperatively in the gas group and at least 3 months after silicone oil removal in the SO group (post-tamponade clearance). This post-clearance time point was considered the definitive anatomical outcome [9].

### Surgical method

All surgeries were performed by the same experienced retinal surgeon using the standard 23 or 25 gauge three-port PPV technique. Perfluorocarbon liquid (PFCL) was used intraoperatively as a routine surgical adjunct in all cases to stabilize the posterior retina and facilitate subretinal fluid drainage during fluid-air exchange. Its uniform application across both treatment groups ensures that any potential retinal effect attributable to PFCL would be equally distributed and would not differentially confound between-group comparisons. No PFCL-related intraoperative or postoperative complications, including retained subretinal PFCL or emulsification, were documented in any patient. Endolaser photocoagulation was applied to retinal tears. Tamponade type was decided by the surgeon case-by-case. Gas tamponade (14% C3F8 or 20% SF6) was preferred in patients with superior and inferior tear locations, single or few tears, and adequate postoperative positioning compliance. Consistent with published practice patterns, SF6 20% was used to tamponade retinal breaks predominantly in the superior quadrant (above the 4–8 o’clock position), while C3F8 14% was used for inferior quadrant breaks; this selection reflects the longer intraocular persistence of C3F8, which provides extended tamponade coverage for inferiorly located tears [10]. Silicone oil (2000 or 5000 cSt) was used in patients with multiple or large tears, giant tears, and poor position adaptation. Although silicone oil is classically indicated for complex cases with advanced PVR, in this cohort silicone oil was also selected in eyes with PVR Grade None or A when other complexity-defining features were present that are not captured by PVR grading alone — specifically: multiple breaks, large or giant retinal tears, inferior tear location requiring prolonged inferior tamponade, and cases in which reliable postoperative head positioning compliance could not be ensured, as well as patients who had planned air travel or had significantly impaired vision in the fellow eye and required early postoperative visual rehabilitation [10]. These clinical considerations drove tamponade selection in a subset of patients without advanced PVR, reflecting real-world surgical decision-making. Silicone oil was surgically removed after an average of 7.8 ± 3.2 months after retinal stabilization was achieved. The relatively prolonged mean silicone oil retention period in this cohort, compared with the typically reported 3–6 months in uncomplicated primary RRD series, is attributable to the higher complexity profile of cases selected for silicone oil tamponade in clinical practice — including eyes with multiple or large tears, giant tears, grade B–C PVR, and greater quadrant involvement. In these cases, extended tamponade was maintained until confirmed retinal stability was achieved at fundus examination, and the decision to remove silicone oil was made individually by the operating surgeon based on clinical criteria rather than a fixed protocol. The extended retention period may have contributed to the greater degree of inner retinal layer thinning observed in the silicone oil tamponade group. In cases deemed necessary, cataract surgery was added to the procedure simultaneously with vitrectomy.

### Statistical analysis

Propensity score matching was employed to minimize baseline group differences and to enable a valid comparison of visual outcomes, retinal thickness measurements, and intraocular pressure changes between tamponade groups. Data analysis was performed using IBM SPSS Statistics (version 26.0). Continuous variables are presented as mean ± SD for normally distributed data and as median (interquartile range [IQR]) for skewed distributions, as assessed by the Shapiro–Wilk test. An independent samples t-test was used for normally distributed parameters, and the Mann–Whitney U test was used for non-normally distributed parameters. The researchers used a paired t-test or Wilcoxon signed-rank test to analyze the changes that occurred within the group. Three time-period measurements in the silicone oil group were analyzed using repeated-measures ANOVA. The Chi-square test examined categorical data, but researchers were required to use Fisher’s exact test when the expected values were less than five.

**Fig. 2:**
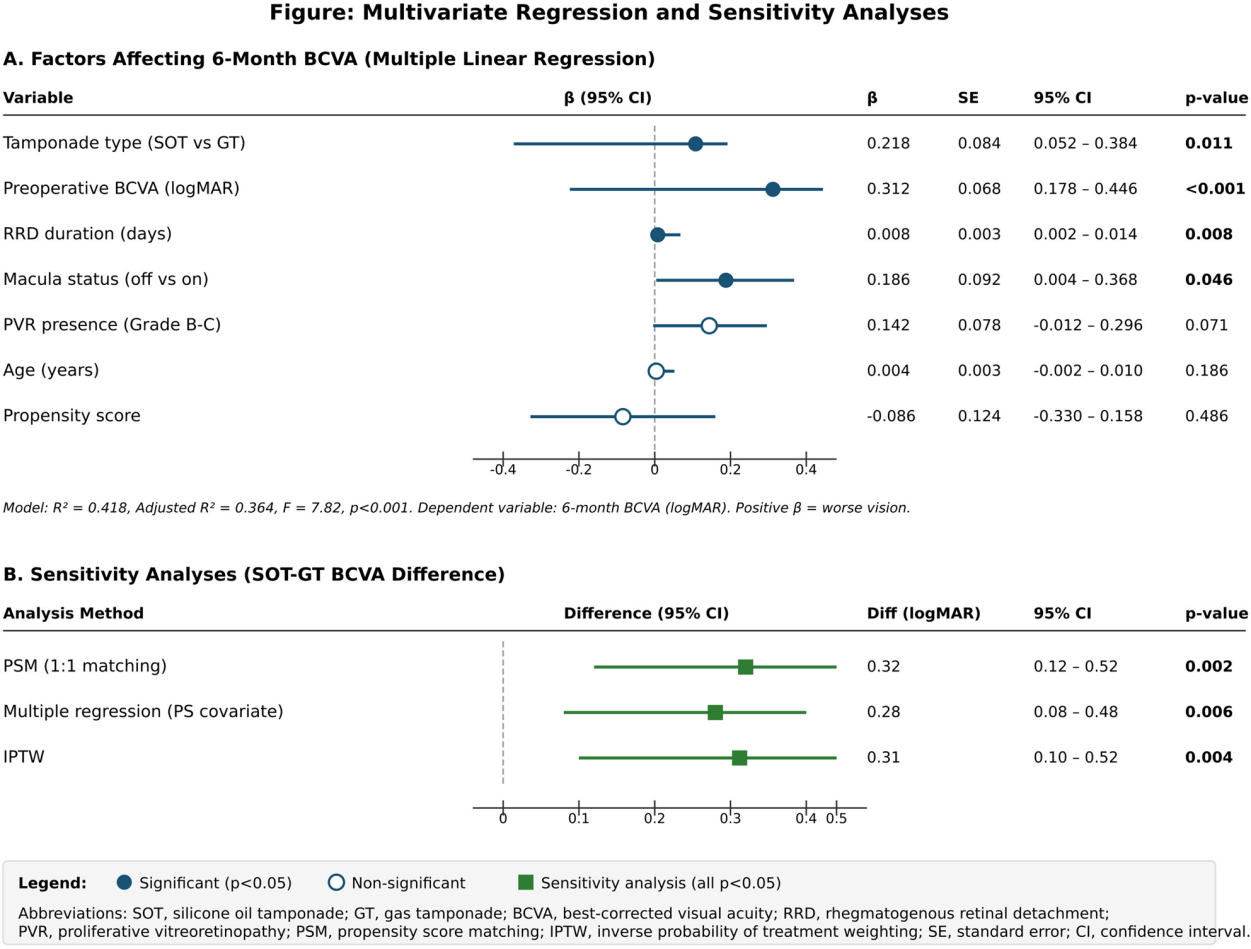
Propensity score matching diagnostics. (**A**) Propensity score distribution before matching. (**B**) Propensity score distribution after matching. (**C**) Covariate balance: standardized mean differences before and after matching; dashed line indicates balance threshold (SMD = 0.20). GT: gas tamponade; SOT: silicone oil tamponade; SMD: standardized mean difference; BCVA: best-corrected visual acuity; IOP: intraocular pressure; PVR: proliferative vitreoretinopathy; RRD: rhegmatogenous retinal detachment

The Propensity Score Matching (PSM) method was applied to minimize the baseline differences between the study groups. The study included age, sex, preoperative visual acuity, IOP, axial length, lens status, detachment duration, macular status, PVR grade, choroidal detachment, and giant tears as covariates. The matching procedure used the 1:1 nearest-neighbor method with a 0.1 caliper width. Adequate covariate balance was defined a priori as a standardized mean difference (SMD) of < 0.20 for all matched covariates. Covariate balance before and after matching was assessed using SMDs (Table 2). The distribution of propensity scores before and after matching is presented in (Fig. 2). Scleral buckling combination, detachment extent (number of quadrants), and RRD etiology were not included as separate covariates in the PSM model because these variables were considered downstream consequences of disease severity rather than independent confounders; their effects were already captured by PVR grade, complex RD classification, and symptom-to-surgery duration. Furthermore, preliminary models incorporating these variables resulted in insufficient overlap in propensity score distributions and poor matching quality.

**Fig. 3:**
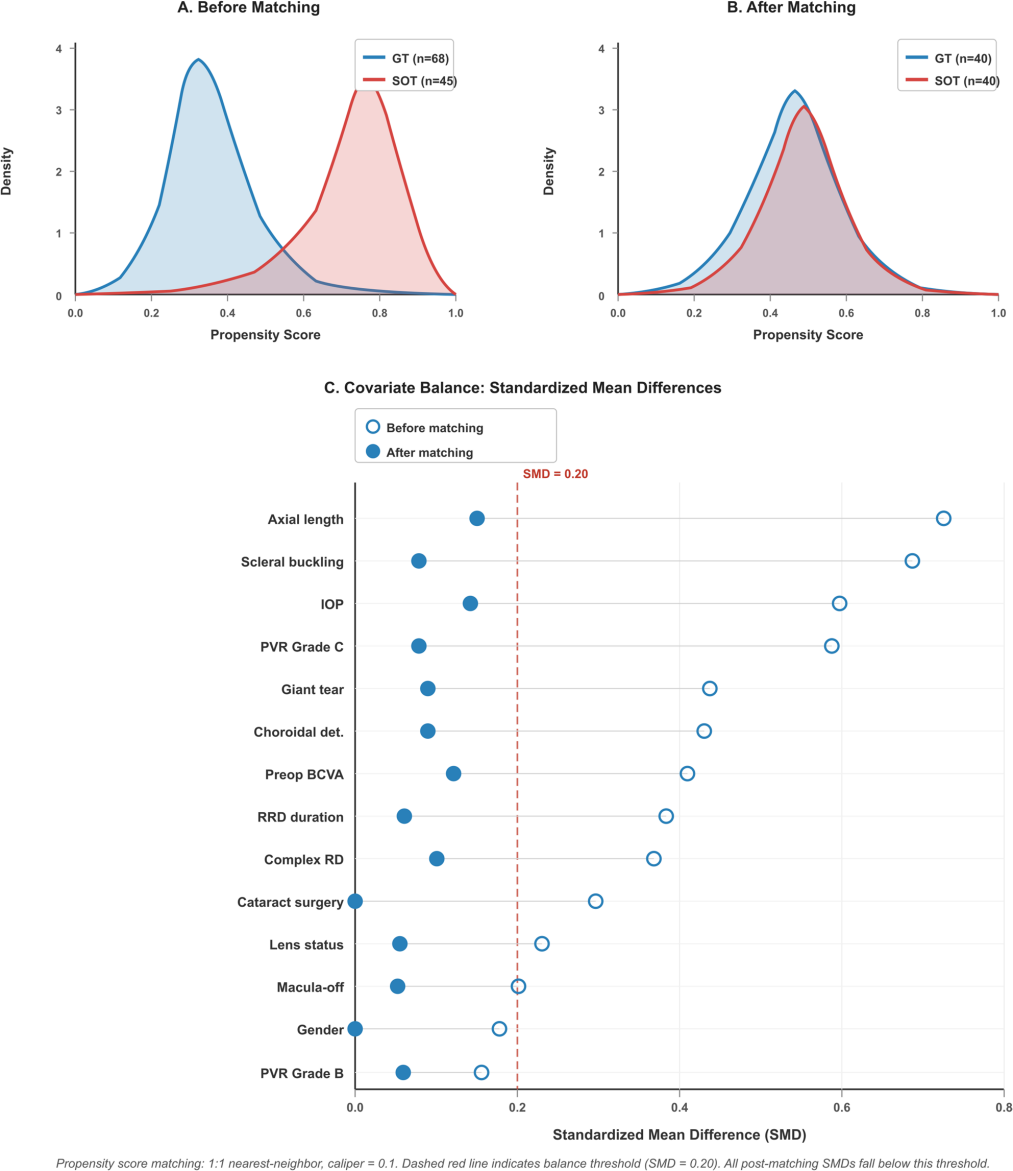
Forest plot of predictors for 6-month visual acuity. (**A**) Multiple linear regression. (**B**) Sensitivity analyses. BCVA, best-corrected visual acuity; SOT, silicone oil tamponade; GT, gas tamponade; PVR, proliferative vitreoretinopathy; PSM, propensity score matching; IPTW, inverse probability of treatment weighting

The Jonckheere–Terpstra trend test evaluated the pattern of detachment duration and visual outcome changes that occurred during the study period. Two-way ANOVA was used to study the interaction between tamponade type and macular status. Multiple linear regression was employed to identify independent predictors of visual acuity at six months. The robustness of the findings was confirmed through sensitivity analyses using multiple regression with propensity score as a covariate and inverse probability of treatment weighting (IPTW) (Fig. 3). Statistical significance was set at *p* < 0.05.

A secondary subgroup analysis comparing functional and anatomical outcomes between patients who received C3F8 gas tamponade and those who received silicone oil was planned, given that C3F8 is more comparable to silicone oil in terms of tamponade duration and the complexity profile of cases in which it is typically indicated, with available evidence showing that both C3F8 and SF6 gas tamponades yield superior BCVA outcomes compared to silicone oil while achieving comparable primary anatomical success rates [10]. However, due to the limited number of patients receiving C3F8 tamponade within this cohort, this comparison lacked adequate statistical power and is therefore acknowledged as a study limitation.

### Ethics approval

This study was approved by the Ethics Committee of Azerbaijan Medical University (Protocol No: AMU/EC-2025-062/O, Date: 26.12.2025). All procedures were conducted in accordance with the ethical standards of the Declaration of Helsinki and good clinical practice guidelines.

### Consent to participate

Due to the retrospective design of this study, the requirement for individual informed consent was waived by the Ethics Committee. All patient data were fully anonymized, and patient privacy was protected at the highest level throughout the study.

## Results

### Patient characteristics and demographic data

A total of 113 eyes were evaluated within the scope of the study, of which 68 (60.2%) underwent gas tamponade and 45 (39.8%) received silicone oil (SO) tamponade. There was no statistically significant difference between the groups in terms of age or sex distribution (*p* > 0.05). However, preoperative baseline evaluation revealed that patients in the SO group had significantly lower visual acuity (median 1.00 vs. 0.70 logMAR, *p* < 0.001), and complicated features such as proliferative vitreoretinopathy (PVR), choroidal detachment, and giant tear were more common in this group. In addition, the symptom-to-surgery duration was approximately twice as long in the SO group than that in the gas group (median 12 vs. 5 days, *p* = 0.019). These data indicate that silicone oil is preferred primarily in cases of more complex retinal detachment in clinical practice (Table 1).

**Table 1:** Demographic and clinical characteristics (Pre-PSM)

Parameters	GT (*n* = 68)	SOT (*n* = 45)	*p*-value
Demographics			
Age, year (mean ± SD)	59.2 ± 12.4	58.7 ± 17.2	0.394ᵃ
Gender, male n (%)	46 (67.6)	34 (75.6)	0.358ᵇ
Preoperative Ocular Findings			
Preoperative BCVA, logMAR, median (IQR)†	0.70 (0.40–1.30)	1.00 (0.52–1.80)	**< 0.001**ᵃ
IOP, mmHg (mean ± SD)	14.6 ± 3.2	12.4 ± 4.1	**0.002**ᵃ
Axial length, mm (mean ± SD)	24.1 ± 1.6	25.8 ± 2.9	**0.018**ᵃ
Lens status, n (%)			0.241ᵇ
— Phakic	41 (60.3)	22 (48.9)	
— Pseudophakic	27 (39.7)	22 (48.9)	
— Aphakic	0 (0)	1 (2.2)	
RRD Features			
Symptom-to-surgery duration, days, median (IQR)†	5 (2–12)	12 (4–30)	**0.019**ᵃ
RRD type, n (%)			**< 0.001**ᵇ
— PVD-related	58 (85.3)	26 (57.8)	
— Atrophic hole	5 (7.4)	3 (6.7)	
— Atopic dermatitis	1 (1.5)	4 (8.9)	
— Myopic macular hole	2 (2.9)	4 (8.9)	
— Traumatic	2 (2.9)	8 (17.8)	
Macular status, n (%)			0.178ᵇ
— Macula-on	34 (50.0)	18 (40.0)	
— Macula-off	34 (50.0)	27 (60.0)	
RRD extent, n (%)			**0.031**ᵇ
— 1 quadrant	16 (23.5)	4 (8.9)	
— 2 quadrants	29 (42.6)	17 (37.8)	
— 3 quadrants	15 (22.1)	13 (28.9)	
— 4 quadrants	8 (11.8)	11 (24.4)	
PVR grade, n (%)			**< 0.001**ᵇ
— None / Grade A	49 (72.1)	20 (44.4)	
— Grade B	15 (22.1)	13 (28.9)	
— Grade C	4 (5.9)	12 (26.7)	
Choroidal detachment, n (%)	4 (5.9)	9 (20.0)	**0.017**ᶜ
Giant tear, n (%)	3 (4.4)	8 (17.8)	**0.022**ᶜ
Surgical Features			
Combined cataract surgery, n (%)	46 (67.6)	24 (53.3)	**0.027**ᵇ
Scleral buckling combination, n (%)	4 (5.9)	14 (31.1)	**< 0.001**ᶜ

ᵃMann–Whitney U test; ᵇChi-square test; ᶜFisher exact test. †Non-normally distributed variables (Shapiro–Wilk *p* < 0.05) are presented as median (IQR). GT: Gas tamponade; SOT: Silicone oil tamponade; PVR: Proliferative vitreoretinopathy

### Propensity score matching (PSM) results

PSM was applied to minimize selection bias that may arise from baseline clinical differences between groups; 40 patients in both groups (80 eyes in total) were included in further analysis. All pre-matching standardized mean differences (SMDs), which ranged from 0.033 to 0.726, were reduced to below the pre-specified balance threshold of 0.20 after matching (all post-matching SMDs ≤ 0.151) (Fig. 2). In the comparison made after matching, intergroup homogeneity was achieved in age, sex, preoperative visual acuity, intraocular pressure (IOP), and axial length parameters (all *p* > 0.05). Similarly, the groups were statistically similar in terms of macular status, PVR grade, and complex detachment rates (Table 2). The overall follow-up period ranged from 6 to 14 months in the gas tamponade group (median 7 months) and from 7 to 18 months in the silicone oil group (median 11 months). The extended follow-up in the SOT group reflects the mean silicone oil retention period of 7.8 ± 3.2 months, after which all patients were followed for a minimum of 3 additional months; total postoperative follow-up therefore exceeded 6 months in all cases, consistent with the inclusion criterion.

**Table 2:** Demographic and clinical characteristics after propensity score matching with covariate balance assessment

Parameters	GT (*n* = 40)	SOT (*n* = 40)	*p*-value	SMD Pre	SMD Post
Age, year (mean ± SD)	58.9 ± 15.8	59.1 ± 16.2	0.847ᵃ	0.033	0.013
Gender, male n (%)	30 (75.0)	30 (75.0)	1.000ᵇ	0.178	0.000
Preop BCVA, logMAR, median (IQR)†	0.80 (0.40–1.40)	0.85 (0.42–1.52)	0.214ᵃ	0.409	0.122
IOP, mmHg (mean ± SD)	15.8 ± 3.4	15.4 ± 2.1	0.762ᵃ	0.598	0.142
Axial length, mm (mean ± SD)	25.2 ± 2.5	25.6 ± 2.8	0.512ᵃ	0.726	0.151
Lens status – Phakic, n (%)	12 (30.0)	11 (27.5)	1.000ᵇ	0.231	0.055
RRD duration, days, median (IQR)†	10 (4–25)	11 (4–28)	0.821ᵃ	0.383	0.061
Macula-off, n (%)	26 (65.0)	27 (67.5)	0.808ᵇ	0.202	0.053
Complex RD, n (%)	20 (50.0)	22 (55.0)	0.654ᵇ	0.369	0.100
Giant tear, n (%)	3 (7.5)	4 (10.0)	1.000ᶜ	0.437	0.089
Choroidal detachment, n (%)	3 (7.5)	4 (10.0)	1.000ᶜ	0.430	0.089
PVR Grade B, n (%)	9 (22.5)	10 (25.0)	0.793ᵇ	0.156	0.059
PVR Grade C, n (%)	4 (10.0)	5 (12.5)	1.000ᶜ	0.587	0.079
Combined cataract surgery, n (%)	23 (57.5)	23 (57.5)	1.000ᵇ	0.296	0.000
Scleral buckling combination, n (%)	4 (10.0)	5 (12.5)	1.000ᶜ	0.687	0.079

ᵃMann–Whitney U test; ᵇChi-square test; ᶜFisher exact test. †Non-normally distributed variables (Shapiro–Wilk *p* < 0.05) are presented as median (IQR). PSM: 1:1 nearest-neighbor matching, caliper = 0.1. SMD: Standardized mean difference; values < 0.20 indicate adequate covariate balance. All post-matching SMDs met this threshold

### Functional and anatomical outcomes

At 1 and 3 months postoperatively, silicone oil was still in situ in the SOT group; therefore, BCVA values at these time points (GT: 0.46 and 0.35 logMAR; SOT: 0.87 and 0.74 logMAR, respectively) are not directly comparable between groups and no between-group p-values are reported. As BCVA data demonstrated non-normal distribution (Shapiro–Wilk *p* < 0.05), median values are presented as the primary comparison metric throughout; mean ± SD values are provided as supplementary measures. According to the postoperative 6-month results, which served as the primary comparison time point, the median BCVA was 0.18 (IQR 0.10–0.40) logMAR in the gas group and 0.48 (IQR 0.22–0.90) logMAR in the SO group (*p* = 0.003); corresponding mean values were 0.32 ± 0.49 and 0.64 ± 0.59 logMAR, respectively. At the 6-month assessment, 28/40 (70%) eyes in the SOT group still had silicone oil in situ (mean SO duration: 7.8 ± 3.2 months). Clinically significant (≥ 2 lines) visual improvement was seen in 70% and 47.5% of the patients in the gas and SO groups, respectively (*p* = 0.042).

To enable a fair comparison after tamponade resolution, a post-clearance analysis was performed. Post-tamponade clearance was defined as the 6-month postoperative assessment in the GT group (gas absorbed within 6–8 weeks) and 3 months after silicone oil removal in the SOT group (mean 10.8 ± 3.2 months postoperatively). Post-clearance median BCVA was 0.18 (IQR 0.10–0.40) logMAR in the gas group and 0.42 (IQR 0.16–0.78) logMAR in the SO group (*p* = 0.038; mean: 0.32 ± 0.49 vs. 0.56 ± 0.52 logMAR), confirming the functional advantage of gas tamponade even after eliminating the confounding effect of silicone oil on visual acuity measurement.

IOP values showed a significant increase in the SO group compared to baseline (within-group *p* = 0.026) and the amount of IOP change was higher than in the gas group (+ 1.4 vs. + 0.3 mmHg, *p* = 0.031). Antiglaucomatous therapy was required in 9 (22.5%) eyes in the SO group versus 4 (10.0%) in the gas group (*p* = 0.128).

Regarding anatomical outcomes, the definition of anatomical success was “retina attached without any tamponade.” Because 70% of SOT eyes still had oil in situ at 6 months, this definition could not be uniformly applied at that time point. Anatomical success at 6 months (with SO in situ serving as a supportive measure) was 95.0% and 92.5% in the GT and SOT groups, respectively (*p* = 1.000). The definitive post-tamponade clearance anatomical success was 95.0% in the GT group and 87.5% in the SOT group (*p* = 0.432). In the SOT group, 3 re-detachments occurred during tamponade and 2 additional re-detachments occurred after silicone oil removal, yielding a total re-detachment rate of 12.5% compared with 5.0% in the GT group (*p* = 0.432). Unexplained vision loss, defined as postoperative BCVA worse than preoperative BCVA despite confirmed retinal reattachment and in the absence of any identifiable structural cause on OCT, was observed in 1 eye (2.5%) in the gas group and 4 eyes (10.0%) in the silicone oil group (*p* = 0.362). Although this difference did not reach statistical significance, the numerically higher rate in the SOT group is consistent with silicone oil-associated inner retinal toxicity and may have contributed to the functional disparity observed between groups. All functional and anatomical outcomes are summarized in Table 3.

**Table 3:** Postoperative functional outcomes, IOP, and complications (Post-PSM)

Parameters	GT (*n* = 40)	SOT (*n* = 40)	*p*-value
Visual Acuity (BCVA, logMAR)			
BCVA 1st month (mean ± SD)	0.46 ± 0.52	0.87 ± 0.58	N/Aᵈ
BCVA 3rd month (mean ± SD)	0.35 ± 0.47	0.74 ± 0.56	N/Aᵈ
SO status at 6 months, n (%)	— (gas absorbed)	28/40 (70.0) oil in situ	—
BCVA 6th month, median (IQR)†	**0.18 (0.10–0.40)**	**0.48 (0.22–0.90)**	**0.003**ᵃ
BCVA 6th month, mean ± SD	0.32 ± 0.49	0.64 ± 0.59	0.003ᵃ
Post-Tamponade Clearance Comparisonᵍ			
BCVA post-clearance, median (IQR)†	**0.18 (0.10–0.40)**	**0.42 (0.16–0.78)**	**0.038**ᵃ
BCVA post-clearance, mean ± SD	0.32 ± 0.49	0.56 ± 0.52	0.038ᵃ
ΔBCVA baseline to post-clearance, median (IQR)†	0.50 (0.20–1.00)	0.40 (0.12–0.80)	0.072ᵃ
≥ 2-line BCVA gain (post-clearance), n (%)	28 (70.0)	22 (55.0)	0.176ᵇ
Post-clearance BCVA ≤ 0.3 logMAR, n (%)	24 (60.0)	16 (40.0)	0.073ᵇ
Post-clearance BCVA > 1.0 logMAR, n (%)	4 (10.0)	8 (20.0)	0.210ᵉ
6th Month Comparison			
ΔBCVA (baseline – 6th month), median (IQR)†	0.50 (0.20–1.00)	0.30 (0.10–0.70)	**0.012**ᵃ
≥ 2-line BCVA gain (6th month), n (%)	28 (70.0)	19 (47.5)	**0.042**ᵇ
6th month BCVA ≤ 0.3 logMAR, n (%)	24 (60.0)	14 (35.0)	**0.026**ᵇ
6th month BCVA > 1.0 logMAR, n (%)	4 (10.0)	11 (27.5)	**0.048**ᵉ
Intraocular Pressure (IOP, mmHg)			
Preoperative IOP (mean ± SD)	15.8 ± 3.4	15.4 ± 2.1	0.762ᵃ
6th month IOP (mean ± SD)	16.1 ± 3.6	16.8 ± 2.4	0.089ᵃ
ΔIOP (mean ± SD)	**+ 0.3 ± 1.8**	**+ 1.4 ± 2.2**	**0.031**ᵃ
Within-group IOP change p-value	0.564ᶜ	**0.026**ᶜ	—
Anatomical Outcomes and Complications			
Anatomical success at 6 months (SO in situ), n (%)ʰ	38 (95.0)	37 (92.5)	1.000ᵉ
Anatomical success post-tamponade clearance, n (%)ᵍ	**38 (95.0)**	**35 (87.5)**	0.432ᵉ
Total re-detachment, n (%)	2 (5.0)	5 (12.5)	0.432ᵉ
Re-detachment during tamponade, n (%)	2 (5.0)	3 (7.5)	1.000ᵉ
Re-detachment after SO removal, n (%)	—	2 (5.0)	—
Unexplained vision loss, n (%)ⁱ	1 (2.5)	4 (10.0)	0.362ᵉ
Antiglaucomatous therapy, n (%)	4 (10.0)	9 (22.5)	0.128ᵉ
ERM development, n (%)	3 (7.5)	5 (12.5)	0.712ᵉ
CME development, n (%)	2 (5.0)	4 (10.0)	0.675ᵉ
Cataract progression (phakic eyes), n (%)	4/12 (33.3)	6/11 (54.5)	0.414ᵉ

ᵃMann–Whitney U test; ᵇChi-square test; ᶜPaired t-test or Wilcoxon signed-rank test; ᵉFisher exact test. †Non-normally distributed variables (Shapiro–Wilk *p* < 0.05) are presented as median (IQR). ᵈBetween-group comparison not applicable at 1 and 3 months, as silicone oil remained in situ in the SOT group. ᵍPost-tamponade clearance: 6 months postoperatively in the GT group (gas absorbed by 6–8 weeks); 3 months after silicone oil removal in the SOT group (mean removal at 10.8 ± 3.2 months). ʰAt 6 months, 28/40 (70%) SOT eyes had silicone oil in situ (mean duration 7.8 ± 3.2 months); the tamponade-free definition of anatomical success therefore could not be uniformly applied at this time point. ⁱDefined as postoperative BCVA worse than baseline despite confirmed reattachment and no structural cause on OCT. Follow-up: GT median 7 months (range 6–14); SOT median 11 months (range 7–18). ERM: Epiretinal membrane; CME: Cystoid macular edema

### Optical coherence tomography (OCT) Data

Prior to assessing postoperative OCT changes, baseline OCT parameters were compared between the two matched groups. No statistically significant differences were identified in preoperative CMT, RNFL, GCL, GCL + IPL thickness, or any parafoveal quadrant thickness parameter between groups (all *p* > 0.05), confirming structural baseline comparability. A statistically significant difference was noted in preoperative retinal volume (8.8 ± 1.2 vs. 7.9 ± 1.2 mm³; *p* = 0.003) and subfoveal choroidal thickness (245 ± 37 vs. 218 ± 68 μm; *p* = 0.031), which is consistent with the marginally greater axial length in the SOT group and does not affect the validity of inner retinal layer outcome comparisons (Table 4D).

OCT analysis showed that all parafoveal quadrants (nasal, temporal, superior, and inferior) experienced significant retinal thinning following the administration of silicone oil tamponade (*p* < 0.001). Notably, inner retinal layers demonstrated significant thinning in the SO group: RNFL thickness decreased from 36 ± 7 μm preoperatively to 28 ± 6 μm during SO tamponade (*p* < 0.001), GCL thickness from 42 ± 8 to 33 ± 7 μm (*p* < 0.001), and GCL + IPL complex from 76 ± 11 to 61 ± 10 μm (*p* < 0.001). The removal of silicone oil led to some improvement in these layers (RNFL: 32 ± 6, GCL: 37 ± 7, GCL + IPL: 68 ± 10 μm), but the measured values remained below the original preoperative values. In the gas group, it was observed that the central macular thickness and inner retinal layers were better preserved, and there was no significant change in the parafoveal regions (RNFL: 37 vs. 36 μm, *p* = 0.486; GCL: 43 vs. 42 μm, *p* = 0.524; GCL + IPL: 78 vs. 77 μm, *p* = 0.612). Between-group comparison at the definitive time point (GT 6th month vs. SOT post-SO removal) revealed significantly thinner RNFL (32 vs. 36 μm, *p* = 0.008), GCL (37 vs. 42 μm, *p* = 0.004), and GCL + IPL (68 vs. 77 μm, *p* < 0.001) in the SO group. The 6-month assessments also showed that the gas group had higher measurements of central macular thickness, total retinal thickness, and subfoveal choroidal thickness. Qualitative evaluation showed that foveal contour flattening occurred more often in the SO group at a rate of 15% compared to the gas group, which showed this effect at 2.5% (*p* = 0.048). RNFL wedge-shaped defects were also more frequent in the SO group (20.0% vs. 5.0%, *p* = 0.043) (Table 4).

**Table 4:** Retinal and Choroidal Thickness Changes with OCT

A. Silicone Oil Group (*n* = 40) – Repeated Measurements				
Parameters (µm)	Preoperative	SO in situ	Post-SO removal	pᵃ
CMT (mean ± SD)	285 ± 58	256 ± 31	272 ± 29	0.072
Nasal thickness (mean ± SD)	344 ± 32	308 ± 28	322 ± 27	**< 0.001**
Temporal thickness (mean ± SD)	326 ± 35	294 ± 31	308 ± 26	**< 0.001**
Superior thickness (mean ± SD)	339 ± 25	306 ± 29	319 ± 24	**< 0.001**
Inferior thickness (mean ± SD)	336 ± 33	304 ± 30	318 ± 29	**< 0.001**
RNFL thickness (mean ± SD)	36 ± 7	28 ± 6	32 ± 6	**< 0.001**
GCL thickness (mean ± SD)	42 ± 8	33 ± 7	37 ± 7	**< 0.001**
GCL + IPL thickness (mean ± SD)	76 ± 11	61 ± 10	68 ± 10	**< 0.001**
Inner retinal thickness (mean ± SD)	129 ± 16	124 ± 35	134 ± 31	0.348
Total retinal thickness (mean ± SD)	236 ± 71	216 ± 42	226 ± 34	0.224
Retinal volume, mm³ (mean ± SD)	7.9 ± 1.2	6.4 ± 1.0	7.7 ± 1.1	**< 0.001**
SFCT (mean ± SD)	218 ± 68	198 ± 71	204 ± 64	0.112

B. Gas Tamponade Group (*n* = 40) – Pre-Post Comparison				
Parameters (µm)	Preoperative	Postop 6th month	*p*	
CMT (mean ± SD)	279 ± 27	291 ± 29	**0.002**ᵇ	
Nasal thickness (mean ± SD)	348 ± 22	347 ± 24	0.542ᶜ	
Temporal thickness (mean ± SD)	333 ± 18	334 ± 17	0.118ᶜ	
Superior thickness (mean ± SD)	346 ± 19	343 ± 21	0.964ᶜ	
Inferior thickness (mean ± SD)	346 ± 27	345 ± 23	0.836ᵇ	
RNFL thickness (mean ± SD)	37 ± 6	36 ± 7	0.486ᶜ	
GCL thickness (mean ± SD)	43 ± 7	42 ± 8	0.524ᵇ	
GCL + IPL thickness (mean ± SD)	78 ± 10	77 ± 11	0.612ᵇ	
Inner retinal thickness (mean ± SD)	133 ± 24	148 ± 31	**0.003**ᵇ	
Total retinal thickness (mean ± SD)	226 ± 24	240 ± 31	**0.003**ᵇ	
Retinal volume, mm³ (mean ± SD)	8.8 ± 1.2	8.4 ± 1.1	0.276ᶜ	
SFCT (mean ± SD)	245 ± 37	239 ± 46	0.251ᶜ	

C. Between-Group Comparison (GT: 6th Month vs. SOT: Post-SO Removal)				
Parameters	GT (*n* = 40)	SOT (*n* = 40)	*p*ᵈ	
Quantitative OCT				
CMT, µm (mean ± SD)	291 ± 29	272 ± 29	**0.004**	
RNFL thickness, µm (mean ± SD)	36 ± 7	32 ± 6	**0.008**	
GCL thickness, µm (mean ± SD)	42 ± 8	37 ± 7	**0.004**	
GCL + IPL thickness, µm (mean ± SD)	77 ± 11	68 ± 10	**< 0.001**	
Inner retinal thickness, µm (mean ± SD)	148 ± 31	134 ± 31	**0.018**	
Total retinal thickness, µm (mean ± SD)	240 ± 31	226 ± 34	**0.028**	
Retinal volume, mm³ (mean ± SD)	8.4 ± 1.1	7.7 ± 1.1	**0.006**	
SFCT, µm (mean ± SD)	239 ± 46	204 ± 64	**0.012**	
Qualitative OCT				
Foveal contour flattening, n (%)	1 (2.5)	6 (15.0)	**0.048**ᵉ	
Ellipsoid zone disruption, n (%)	3 (7.5)	8 (20.0)	0.102ᵉ	
IS/OS band discontinuity, n (%)	4 (10.0)	10 (25.0)	0.082ᵉ	
RNFL wedge-shaped defect, n (%)	2 (5.0)	8 (20.0)	**0.043**ᵉ	
Subfoveal fluid, n (%)	1 (2.5)	3 (7.5)	0.615ᵉ	
Cystoid macular changes, n (%)	3 (7.5)	7 (17.5)	0.182ᵉ	

D. Preoperative Between-Group OCT Comparison (Post-PSM Matched Groups)				
Parameters (µm)	GT (*n* = 40) Preoperative	SOT (*n* = 40) Preoperative	*p*ᶠ	
Foveal / Central				
CMT (mean ± SD)	279 ± 27	285 ± 58	0.562	
Parafoveal Quadrant Thickness				
Nasal (mean ± SD)	348 ± 22	344 ± 32	0.524	
Temporal (mean ± SD)	333 ± 18	326 ± 35	0.261	
Superior (mean ± SD)	346 ± 19	339 ± 25	0.164	
Inferior (mean ± SD)	346 ± 27	336 ± 33	0.148	
Inner Retinal Layers				
RNFL thickness (mean ± SD)	37 ± 6	36 ± 7	0.512	
GCL thickness (mean ± SD)	43 ± 7	42 ± 8	0.598	
GCL + IPL thickness (mean ± SD)	78 ± 10	76 ± 11	0.402	
Inner retinal thickness (mean ± SD)	133 ± 24	129 ± 16	0.388	
Overall Retinal Parameters				
Total retinal thickness (mean ± SD)	226 ± 24	236 ± 71	0.406	
Retinal volume, mm³ (mean ± SD)	8.8 ± 1.2	7.9 ± 1.2	**0.003**ᶠ	
Choroid				
SFCT (mean ± SD)	245 ± 37	218 ± 68	**0.031**ᶠ	

ᵃRepeated-measures ANOVA

ᵇPaired t-test; ᶜWilcoxon signed-rank test

ᵈMann–Whitney U test; ᵉFisher exact test

ᶠMann–Whitney U test. Panel D presents preoperative between-group OCT comparisons in the PSM-matched cohort to confirm structural baseline equivalence. For panels B–C, GT values represent the 6-month postoperative assessment (gas absorbed by 6–8 weeks); SOT values represent the post-removal assessment (≥ 3 months after removal; mean SO duration 7.8 ± 3.2 months). CMT: Central macular thickness; SFCT: Subfoveal choroidal thickness; RNFL: Retinal nerve fiber layer; GCL: Ganglion cell layer; IPL: Inner plexiform layer; IS/OS: Inner segment/outer segment

### Subgroup and interaction analyses

In the analysis based on the duration of detachment, visual acuity worsened in both groups as the duration increased (Jonckheere–Terpstra trend test: GT *p* = 0.018, SOT *p* = 0.006). However, the functional superiority of the gas group was maintained in all time periods, including detachments of less than 3 days. Based on macular status, the difference between the groups was not significant in the macula-on cases (*p* = 0.064), while there was a significant difference in favor of gas tamponade in the macula-off cases (*p* = 0.006). In addition, the type of tamponade and macular status interaction was significant (*p* = 0.042), indicating that the disadvantage of silicone oil tamponade was disproportionately greater in macula-off eyes (difference: 0.38 logMAR) compared with macula-on eyes (difference: 0.18 logMAR). Regardless of the presence of PVR, better visual results were obtained in the gas group, whereas anatomical success at post-tamponade clearance was similar in both groups (Table 5).

**Table 5:** Subgroup Analysis (6th Month BCVA, logMAR)

A. By RRD Duration						
RRD Duration		GT *n*	GT BCVA	SOT *n*	SOT BCVA	pᵃ
≤ 3 days		12	0.21 ± 0.28	8	0.42 ± 0.38	**0.048**
4–7 days		16	0.28 ± 0.36	14	0.58 ± 0.52	**0.024**
≥ 8 days		12	0.48 ± 0.62	18	0.84 ± 0.71	**0.031**
p (trend)ᵇ			**0.018**		**0.006**	
**B. By Macular Status**						
**Macula**		**n (GT/SOT)**	**GT BCVA**	**SOT BCVA**	**Difference**	**p**ᵃ
Macula-on		14/13	0.14 ± 0.18	0.32 ± 0.26	0.18	0.064
Macula-off		26/27	0.42 ± 0.54	0.80 ± 0.62	0.38	**0.006**
p (macular effect)ᶜ			**0.018**	**0.004**		
p (interaction)ᵈ						**0.042**
**C. By PVR Status**						
**PVR**	**n (GT/SOT)**	**GT BCVA**	**SOT BCVA**	**Anatomical Success GT**	**Anatomical Success SOT**	**p**ᵃ **(BCVA)**
None/A	27/25	0.25 ± 0.34	0.52 ± 0.46	26 (96.3%)	24 (96.0%)	**0.024**
B	9/10	0.40 ± 0.52	0.78 ± 0.62	9 (100%)	8 (80.0%)	**0.044**
C	4/5	0.58 ± 0.74	1.02 ± 0.80	3 (75.0%)	3 (60.0%)	0.142

ᵃMann–Whitney U test; ᵇJonckheere–Terpstra trend test; ᶜMann–Whitney U (within-group comparison); ᵈTwo-way ANOVA (tamponade × macular status interaction). Anatomical success refers to post-tamponade clearance as defined in Table 3. PVR Grade C subgroup analysis is limited by small sample sizes (GT *n* = 4, SOT *n* = 5)

Among the 40 gas-treated eyes in the matched cohort, 14 (35.0%) received C3F8 14% and 26 (65.0%) received SF6 20% tamponade, selected according to the break location criteria described in the Surgical Method section. Due to the limited number of C3F8-treated eyes (*n* = 14), a statistically powered inferential comparison with the silicone oil group was not feasible. Descriptively, the 6-month mean BCVA in the C3F8 subgroup was 0.40 ± 0.56 logMAR, and post-tamponade clearance anatomical success was 92.9% (13/14 eyes). The SF6 subgroup (*n* = 26) demonstrated a 6-month mean BCVA of 0.28 ± 0.44 logMAR with a post-tamponade clearance anatomical success of 96.2% (25/26 eyes). The numerically higher BCVA in the C3F8 subgroup compared with the SF6 subgroup is consistent with the more complex break profile (predominantly inferior location) for which C3F8 was selected. Both gas subgroups remained functionally superior to the silicone oil group (mean BCVA 0.56 ± 0.52 logMAR post-clearance), supporting the functional advantage of gas tamponade regardless of gas agent type. These exploratory findings are hypothesis-generating only and should be interpreted in the context of the small C3F8 sample size; a dedicated prospective comparison is warranted.

### Multivariate regression and sensitivity analysis

The regression model identified tamponade type, baseline visual acuity, detachment duration, and macular status as independent risk factors that affected visual acuity at the sixth month. The use of silicone oil resulted in worse visual outcomes, which did not appear to depend on other confounding factors. In all PSM, multiple regression, and IPTW (inverse probability of treatment weighting) analyses performed to test the robustness of the findings, a lower visual performance in the range of 0.28–0.32 logMAR was detected in the silicone oil group, confirming the methodologically consistent results (Fig. 3).

## Discussion

The choice of tamponade in primary rhegmatogenous retinal detachment surgery is a key factor that directly determines surgical success. Although gas and silicone oil options have been used in clinics for a long time, the question of which tamponade offers superior outcomes remains a subject of ongoing debate. In this study, which was conducted with propensity score matching, it was observed that gas tamponade was superior in functional outcomes, while retinal thinning and increased intraocular pressure (IOP) were prominent in the silicone oil group. Especially in cases of macular involvement, tamponade preference has been found to affect not only anatomical healing, but also long-term visual quality. Importantly, because a substantial proportion of silicone oil eyes still had oil in situ at 6 months, a post-tamponade clearance analysis was performed to ensure a fair comparison after complete tamponade resolution in both groups.

As BCVA data demonstrated non-normal distribution (Shapiro–Wilk *p* < 0.05), median values are presented as the primary comparison metric throughout. In the present study, the median 6-month BCVA was 0.18 (IQR 0.10–0.40) logMAR in the gas group and 0.48 (IQR 0.22–0.90) logMAR in the silicone oil group (*p* = 0.003); corresponding mean values were 0.32 ± 0.49 and 0.64 ± 0.59 logMAR, respectively. These results are in agreement with the data of Liu et al., who reported levels of 0.17 logMAR in the gas group and 0.49 logMAR in the silicone oil group [11]. At early postoperative time points, silicone oil remained in situ in the SOT group, precluding between-group VA comparison due to the refractive confounding effect of silicone oil (refractive index ~ 1.40). The persistent functional advantage of gas tamponade at the post-clearance time point (median 0.18 [IQR 0.10–0.40] vs. 0.42 [IQR 0.16–0.78] logMAR, *p* = 0.038) indicates that the observed difference reflects a genuine disparity in visual recovery rather than an artifact of silicone oil’s refractive properties. At least two lines of visual improvement were observed in 70% of the gas group and 47.5% of the silicone oil group at 6 months (*p* = 0.042). The definitive post-tamponade clearance anatomical success was 95.0% in the GT group and 87.5% in the SOT group (*p* = 0.432), with 2 additional re-detachments occurring after silicone oil removal in the SOT group. IOP elevation was more pronounced in the silicone oil group (within-group *p* = 0.026; ΔIOP: +1.4 ± 2.2 vs. + 0.3 ± 1.8 mmHg, *p* = 0.031), consistent with the reported 2.1–56% prevalence of SO-induced IOP rise in the literature [12] and the elevated IOP rates documented in SO-treated eyes by Liu et al [11]. In the present series, unexplained vision loss — defined as postoperative BCVA worse than preoperative values despite confirmed retinal reattachment and in the absence of any identifiable structural cause on OCT — was observed in 1 of 40 eyes (2.5%) in the gas group and 4 of 40 eyes (10.0%) in the silicone oil group (*p* = 0.362; Table 3); when restricted to anatomically successful eyes, rates were 1/38 (2.6%) and 4/35 (11.4%), respectively. Although this difference did not reach statistical significance, likely due to limited sample size, the numerically higher rate in the SOT group is consistent with published data: in a large-scale comparative study of 1,012 primary RRD eyes across multiple tamponade agents, SO 1000cs and SO 5000cs were associated with unexplained visual loss rates of 11.6% and 16.7%, respectively, compared with 0.8% for C3F8, and silicone oil remained an independent risk factor for unexplained visual loss on multivariable logistic regression even after adjusting for case complexity, macula status, giant retinal tears, and other confounders [13]. Notably, that study did not incorporate IOP as a covariate in its regression model; the link between SO-induced IOP elevation and ganglion cell toxicity observed in our series — consistent with Pakravan et al. (10.3%) [14] — may therefore represent an additional mechanistic pathway not fully captured in prior analyses. Prolonged SO tamponade may amplify both IOP-mediated and direct retinal toxicity effects [15].

The SO tamponade procedure showed that all areas within the parafoveal quadrants became significantly thinner. Inner retinal layers demonstrated pronounced thinning under SO tamponade: RNFL thickness decreased from 36 ± 7 μm to 28 ± 6 μm (*p* < 0.001), GCL thickness from 42 ± 8 to 33 ± 7 μm (*p* < 0.001), and GCL + IPL complex from 76 ± 11 to 61 ± 10 μm (*p* < 0.001). Partial recovery was observed after SO removal (RNFL: 32 ± 6, GCL: 37 ± 7, GCL + IPL: 68 ± 10 μm), but values remained below preoperative levels. These findings are consistent with Lee et al., who demonstrated that parafoveal GC-IPL thickness showed recovery at 3–6 months after SO removal; however, peripapillary RNFL thickness did not recover even 6 months after removal, suggesting that peripapillary structures may be more vulnerable to permanent SO-related damage [16]. In the between-group comparison, SO eyes had significantly thinner RNFL (32 vs. 36 μm, *p* = 0.008), GCL (37 vs. 42 μm, *p* = 0.004), and GCL + IPL (68 vs. 77 μm, *p* < 0.001) compared with gas eyes, whereas the gas group showed no significant change in any of these layers (all *p* > 0.4). Prior to these postoperative comparisons, baseline comparability of primary inner retinal layer parameters (RNFL, GCL, GCL + IPL) between matched groups was confirmed (all *p* > 0.05); retinal volume and SFCT differed significantly at baseline (*p* = 0.003 and *p* = 0.031, respectively), likely attributable to the marginally greater axial length in the SOT group, but this does not compromise the primary outcome analysis (Table 4D). This layer-specific vulnerability aligns with the review by Christou et al., who attributed the inner retinal changes to multiple mechanisms including reduced oxygen transfer, disruption of electrolyte homeostasis, and direct mechanical compression [17]. Foveal contour flattening was 15% and 2.5% in the SO and gas groups, respectively (*p* = 0.048). RNFL wedge-shaped defects were also significantly more frequent in the SO group (20.0% vs. 5.0%, *p* = 0.043). Wang et al. found a 1.3% increase in peripapillary capillary density after SO removal and attributed this to the disappearance of mechanical compression [18].

In subgroup analyses, the functional advantage of gas tamponade was confined to macula-off cases (*p* = 0.006), with a significant tamponade × macular status interaction (*p* = 0.042), suggesting a synergistic negative effect of SO toxicity and prolonged macular detachment on visual recovery. Even in early-stage cases (< 3 days detachment), gas tamponade remained superior (*p* = 0.048). Yorston et al. proved that detachment duration is the most critical and modifiable factor determining visual recovery in large cohort studies covering 2074 macular-off cases [19]. Hostovsky et al. also stated that foveal structural changes determine the prognosis in macular-off cases [20].

Gas showed better functional results across all PVR grades. However, at PVR-C, although gas (0.58 logMAR) showed numerically better results than silicone oil (1.02 logMAR), the difference did not reach statistical significance (*p* = 0.142), likely due to the small sample sizes in this subgroup (GT *n* = 4, SOT *n* = 5). Post-tamponade clearance anatomical success in PVR-C was 75.0% in the gas group and 60.0% in the SO group. Although Deiss et al. did not find a difference in vision between tamponades at 12 months in the presence of mild-to-moderate PVR, they emphasized that PVR negatively affected the overall prognosis [21]. In the review of Siwik et al., it was reported that silicone oil should be preferred in complex PVR cases, as it provides a more stable tamponade effect and is associated with lower retinal displacement rates compared with gas [22]. The use of silicone oil in eyes with PVR Grade None or A (20 of 45 SOT eyes pre-matching) warrants comment. Although PVR grade is the most commonly cited indication for silicone oil, clinical decision-making in this cohort encompassed additional complexity-defining features not captured by PVR grading alone — specifically: multiple breaks, large or giant retinal tears, inferior tear location requiring prolonged inferior tamponade, and cases in which reliable postoperative head positioning compliance could not be ensured. These clinical considerations reflect real-world surgical decision-making in a tertiary vitreoretinal referral center, consistent with published practice patterns [10].

The clinical relevance of directly comparing C3F8 and silicone oil outcomes merits consideration, given that C3F8 may be more comparable to silicone oil in terms of the complexity profile of cases in which it is selected. In a recent retrospective study of 151 RRD patients comparing SF6, C3F8, and silicone oil tamponades, primary anatomical success rates were similar across groups (SF6 91.9%, C3F8 97.6%, silicone oil 90.4%; *p* = 0.361); however, 12-month BCVA was significantly worse in the silicone oil group (0.91 logMAR) compared with both C3F8 (0.70 logMAR; *p* = 0.041) and SF6 (0.61 logMAR; *p* = 0.004), and secondary glaucoma occurred exclusively in the silicone oil group (8.2%; *p* = 0.036). Notably, propensity score matching was not applied in that study, and the silicone oil group harbored significantly higher rates of initial PVR (19.2% vs. 2.3–2.7%; *p* = 0.004), limiting causal inference [10]. In the present cohort, a dedicated C3F8 versus silicone oil subgroup analysis was not feasible due to the limited number of C3F8-treated eyes; this constitutes a study limitation and warrants prospective investigation.

The preoperative visual acuity score (BCVA) was the most important factor that predicted success (β = 0.312, *p* < 0.001). Zgolli et al. also accepted a baseline vision of 2 logMAR and above as an independent risk factor for poor prognosis [23]. Chereji et al. confirmed in their systematic review that preoperative BCVA, symptom duration, and macular status are the primary determinants of postoperative visual recovery in RRD surgery [24]. The analysis established that the symptom-to-surgery duration was a crucial prognostic factor (β = 0.008, *p* = 0.008), and Zgolli et al. demonstrated that symptoms lasting > 15 days are associated with negative treatment results [23]. The researchers found that tamponade type served as a direct factor that influenced the treatment results (β = 0.218, *p* = 0.011). Our model explained 41.8% of the variance (R²=0.418). The three sensitivity analyses (PSM, multiple regression, and IPTW) generated consistent results, which validated the robustness of the study.

This study has several limitations and strengths. Regarding limitations, first, its retrospective design introduces inherent selection bias; although propensity score matching was applied, scleral buckling combination, detachment extent, and RRD etiology were not included as PSM covariates, as these were considered downstream consequences of disease severity already captured by PVR grade, complex RD classification, and symptom-to-surgery duration. Residual confounding from these unmeasured variables cannot be entirely excluded. Second, the heterogeneous timing of silicone oil removal (mean 7.8 ± 3.2 months) introduces variability in the post-clearance assessment time point; this prolonged retention period, while attributable to the higher complexity profile of selected cases, may have contributed to the greater degree of inner retinal layer thinning observed in the SOT group. Third, because 70% of SOT eyes still had silicone oil in situ at 6 months, the 6-month BCVA comparison may be influenced by the optical properties of silicone oil, although the post-tamponade clearance analysis was designed to account for this. Fourth, preoperative OCT in macula-off eyes may be limited by subretinal fluid artifact, particularly for central foveal parameters; baseline retinal volume and subfoveal choroidal thickness also differed significantly between groups despite matching, likely reflecting the marginally greater axial length in the SOT group, though primary inner retinal layer parameters were comparable. Fifth, the single-center design limits generalizability. Sixth, a dedicated C3F8 versus silicone oil subgroup analysis was not feasible due to the limited number of C3F8-treated eyes in this cohort. Regarding strengths, all surgeries were performed by a single experienced surgeon, ensuring technical standardization; layer-specific OCT analysis (RNFL, GCL, GCL + IPL) was systematically performed with baseline comparability confirmed; and three independent sensitivity analysis methods (PSM, multiple regression, IPTW) yielded consistent results, supporting the robustness of findings. Randomized controlled prospective trials are needed to study the consequences of selecting tamponades for future use, and prospective designs with standardized SO removal timing and dedicated C3F8 versus silicone oil comparisons would further strengthen the evidence base.

## Conclusion

This study demonstrates that gas tamponade offers superior visual outcomes compared to silicone oil in primary rhegmatogenous retinal detachment surgery, both at 6 months (median BCVA 0.18 vs. 0.48 logMAR, *p* = 0.003) and after tamponade clearance (median BCVA 0.18 vs. 0.42 logMAR, *p* = 0.038). Although both tamponade methods exhibited similar anatomical success rates, significant differences were observed in their functional recovery levels. The use of silicone oil has been found to be directly associated with inner retinal layer thinning particularly of the RNFL, GCL, and GCL + IPL complex and a tendency to increase intraocular pressure. A numerically higher rate of unexplained vision loss was also observed in the silicone oil group (10.0% vs. 2.5%), consistent with silicone oil-associated inner retinal toxicity reported in the literature. Especially in cases of macular involvement, tamponade preference is of vital importance. When deciding on tamponade, physicians should not only focus on anatomical success but should also consider long-term visual functions and the potential for silicone oil–related retinal toxicity. Future prospective studies with standardized silicone oil removal timing and dedicated C3F8 versus silicone oil comparisons are warranted to further guide individualized tamponade selection. Appropriate patient selection and individualized treatment approaches play major roles in achieving optimal outcomes.

## Data Availability

Data used in this study can be provided upon reasonable request.
